# Association of apoptosis-related variants to malaria infection and parasite density in individuals from the Brazilian Amazon

**DOI:** 10.1186/s12936-023-04729-6

**Published:** 2023-10-04

**Authors:** Camille Sena-dos-Santos, Giovanna C. Cavalcante, Diego Marques, Caio S. Silva, Milene Raiol de Moraes, Pablo Pinto, Mayara Natália Santana-da-Silva, Rafaella S. Ferraz, Sheyla Patrícia T. Costa, Ana Maria R. Ventura, Marinete M. Póvoa, Maristela G. Cunha, Ândrea Ribeiro-dos-Santos

**Affiliations:** 1 Laboratory of Human and Medical Genetics, Program of Genetics and Molecular Biology, Federal University of Pará (UFPA), Belém, Brazil; 2 Laboratory of Dermatoimmunology, Federal University of Pará (UFPA), Marituba, Brazil; 3 Laboratory of Microbiology and Immunology, Federal University of Pará (UFPA), Belém, Brazil; 4 Division of Parasitology, Evandro Chagas Institute (IEC), Ananindeua, Brazil; 5 Program of Oncology and Medical Sciences, Oncology Research Center, Belém, Brazil

**Keywords:** Malaria, *Plasmodium*, Apoptosis, Genetic ancestry, Genetic markers

## Abstract

**Background:**

In malaria infection, apoptosis acts as an important immunomodulatory mechanism that leads to the elimination of parasitized cells, thus reducing the parasite density and controlling immune cell populations. Here, it was investigated the association of INDEL variants in apoptotic genes—rs10562972 (*FAS*), rs4197 (*FADD*), rs3834129 and rs59308963 (*CASP8*), rs61079693 (*CASP9*), rs4647655 (*CASP3*), rs11269260 (*BCL-2*), and rs17880560 (*TP53*)—and the influence of genetic ancestry with susceptibility to malaria and parasite density in an admixed population from the Brazilian Amazon.

**Methods:**

Total DNA was extracted from 126 malaria patients and 101 uninfected individuals for investigation of genetic ancestries and genotypic distribution of apoptosis-related variants by Multiplex PCR. Association analyses consisted of multivariate logistic regressions, considering the following comparisons: (i) DEL/DEL genotype vs. INS/DEL + INS/INS; and (ii) INS/INS vs. INS/DEL + DEL/DEL.

**Results:**

Individuals infected by *Plasmodium falciparum* had significantly higher African ancestry proportions in comparison to uninfected controls, *Plasmodium vivax,* and mixed infections. The INS/INS genotype of rs3834129 (*CASP8*) seemed to increase the risk for *P. falciparum* infection (*P* = 0.038; OR = 1.867; 95% CI 0.736–3.725), while the DEL/DEL genotype presented a significant protective effect against infection by *P. falciparum* (*P* = 0.049; OR = 0.446; 95% CI 0.185–0.944) and mixed infection (*P* = 0.026; OR = 0.545; 95% CI 0.281–0.996), and was associated with lower parasite density in *P. falciparum* malaria (*P* = 0.009; OR = 0.383; 95% CI 0.113–1.295). Additionally, the INS/INS genotype of rs10562972 (*FAS*) was more frequent among individuals infected with *P. vivax* compared to *P. falciparum* (*P* = 0.036; OR = 2.493; 95% CI 1.104–4.551), and the DEL/DEL genotype of rs17880560 (*TP53*) was significantly more present in patients with mono-infection by *P. vivax* than in individuals with mixed infection (*P* = 0.029; OR = 0.667; 95% CI 0.211–1.669).

**Conclusions:**

In conclusion, variants in apoptosis genes are associated with malaria susceptibility and parasite density, indicating the role of apoptosis-related genetic profiles in immune responses against malaria infection.

**Supplementary Information:**

The online version contains supplementary material available at 10.1186/s12936-023-04729-6.

## Background

Malaria is an infection caused by parasites of the genus *Plasmodium*, which are transmitted to humans by *Anopheles* mosquitoes [1]. According to the World Health Organization (WHO), malaria is still among the most prevalent parasitic diseases, with 247 million infections, and accounting for more than 600,000 deaths worldwide in 2021 [2]. In Brazil, the highest malaria burden and frequency is concentrated in the Amazon region [2–4], with the transmission of *Plasmodium falciparum*, *Plasmodium vivax* and *Plasmodium malariae* by mono or mixed infection caused by multiple species of *Plasmodium* [5].

In human malaria, cellular immune response involves the secretion of high levels of pro-inflammatory cytokines, such as tumour necrosis factor (TNF) [6], interferon-gamma (IFN-γ) [7, 8], the release of free radicals such as reactive oxygen species (ROS) [9], and reactive nitrogen species (RNS) [7, 10], in addition to the production of the anti-inflammatory interleukin-10 (IL-10) to prevent excessive inflammation [6, 8].

These immune components can activate apoptosis, a form of regulated cell death (RCD) associated with the immune response against malaria [11–19]. Apoptosis is a genetically coordinated process that occurs via extrinsic or intrinsic pathways, in response to extracellular and intracellular stimuli, respectively. Both pathways culminate in the activation of initiator and executioner caspases [20, 21]. Among the several genes that orchestrate apoptosis, it can be highlighted: (i) *FAS*, *FADD,* and *CASP8* in the extrinsic/death receptor pathway; (ii) *BCL-2* and *CASP9* in the intrinsic/mitochondrial pathway; (iii) *TP53* and *CASP3* in both pathways or the executing phase [21].

Apoptosis is widely considered a non-lytic form of cell death, although it has a dynamic role in the immunity to malaria [19]. For instance, the apoptotic bodies formed during the apoptosis of parasitized cells may be loaded with *Plasmodium* antigens, which are phagocytized by dendritic cells (DCs) that, in turn, present the antigens to CD8^+^ T cells. Failures in this process may contribute to parasite survival, replication in the host, and higher parasite density [12]. On the other hand, apoptosis is the main mechanism of DCs [15], CD8^+^ T cells [11], and CD4^+^ T cells [16] depletion, restricting the immune response to malaria.

Therefore, the regulated expression of the aforementioned genes is crucial for the proper functioning of apoptosis in the immune response. INDEL variants that alter the activity of encoded apoptotic proteins [22] may affect the susceptibility to *Plasmodium* infection and the rates of parasite density. This study aimed to investigate the association between the genotypic distribution of eight INDEL markers of apoptosis with the susceptibility to *P. falciparum*, *P. vivax,* and mixed infection, and the rates of malaria parasite density in a population of the Brazilian Amazon that presents high levels of genetic admixture.

## Methods

### Study population

Our cohort consisted of 126 patients from Belém (51) and Tucuruí (75), who were diagnosed with malaria after examination of their thick blood smear (TBS), from 2006 to 2010. The samples were collected when participants sought the health service to perform the *Plasmodium* test, individuals with a positive result were included in the case group of the study. In the blood collection, a questionnaire was applied to assess demographic and epidemiological data, wherewith all participants were asked about their age, sex and history of malaria exposure.

The control group (CG) was composed of 101 individuals of the general population from Belém with no history of malaria, collected at the Human and Medical Genetics Laboratory in the Federal University of Pará. Both infected individuals and the CG resided in malaria endemic areas and were randomly selected.

As previously reported by our group [5], the malaria patients were subdivided into: (i) 42 individuals were mono-infected with *P. falciparum*; (ii) 26 mono-infected with *P. vivax*; and (iii) 58 individuals with mixed species of *Plasmodium* infection (42 had mixed-infection with *P. vivax* and *P. malariae*, 12 had mixed-infection with *P. vivax* and *P. falciparum*, and four patients were infected by the three species simultaneously).

### DNA extraction and quantification

Peripheral blood samples were collected in tubes containing ethylenediaminetetraacetic acid (EDTA) as an anticoagulant. DNA extraction was based on the phenol–chloroform protocol [23], and DNA samples were stored at − 20 °C until downstream use. The concentration and integrity of DNA were assessed using NanoDrop 1000 spectrophotometer (Thermo Fisher Scientific, Wilmington, DE, USA).

### Molecular malaria diagnosis and parasite density

All infected samples were first diagnosed as positive by TBS, as recommended by the Brazilian Ministry of Health, before proceeding with real-time quantitative PCR (RT-qPCR). Next, malaria parasites were detected by RT-qPCR, based on the amplification of *P. falciparum*, *P. vivax,* and *P. malariae* mitochondrial DNA (mtDNA) following an established method [24]. Parasite density levels were also assessed by RT-qPCR, using the probes previously designed to detect the mtDNA of the parasites [24].

The quantification of parasite density was obtained by the standard curve method. To construct the standard curve, a serial dilution of the genomic DNA in ultra-pure water (DF 10) was performed until the concentration of 1:100,000 in triplicate. The serial dilutions were performed in the AriaMx Real-time PCR System (Agilent Technologies, Santa Clara, CA, USA) in a final volume of 10 µL containing 3.7 µL of water, 5.0 µL of TaqMan Universal PCR Master Mix, 0.3 µL of TaqMan probe and 1.0 µL of DNA. Next, parameters of the qPCR standard curves such as efficiency, coefficient of determination (r^2^), slope, and intercept were evaluated. Standard curve and threshold of detection are shown in Additional file 1: Fig S1 and Additional file 2: Fig S2.

It should be noted that the detection of the threshold cycle (C_T_) and the construction of the standard curve for *P. malariae* were not performed given that all the malaria cases caused by *P. malariae* observed in this study occurred as mixed infections, therefore parasite density of this species could not be reliably estimated by TBS and qPCR.

### Selection of INDEL markers

The panel of the eight apoptotic markers was designed and implemented by Cavalcante and colleagues [22]. Complying with four established criteria: (i) variants must be found in genes involved in apoptosis pathways; (ii) they must be INDEL type variants; (iii) they must have the potential to modify protein expression, including INDELs present in intronic regions; (iv) they must have minor allele frequency (MAF) ≥ 10%. The molecular features of investigated markers are shown in Table 1.

**Table 1:** Characterization of the INDEL variants included in the panel

Genes	ID	Region	Alleles	MAF	Primers	Amplicon size
*FAS*	rs10532972	Intron	TTC/-	0.12	F5’GCATCAGGACGCTGAACATA3’ R5’AATGCAACTTGCTCCAGAGG3’	368–371
*FADD*	rs4197	3’-UTR	-/TGT	0.47	F 5’TGCCCCTACTTAGCAGTCTCA3’ R 5’GAGAGGTGGAGAACTGGGATT3’	278–281
*CASP8*	rs3834129	Promoter	AGTAAG/-	0.39	F 5’CTCTTCAATGCTTCCTTGAGGT3’ R 5’CTGCATGCCAGGAGCTAAGTAT3’	249–255
*CASP8*	rs59308963	Intron	ATTCTGTC/-	0.26	F 5’TTTTTGTCCTCCAAGCTTCC3’ R 5’GAACAAGAGAGAGGGCAGGA3’	261–269
*CASP9*	rs61079693	Intron	AAAA/-	0.32	F 5’CATGCACAGCTATCCAGGAG3’ R 5’TTGTTCCTGTCCGATAGATGC3’	458–462
*CASP3*	rs4647655	Intron	-/AAATCCTGAA	0.28	F 5’AGGAGTATCCCCTCGTGGAC3’ R 5’CAAGAGTCAGGCAAAAACAGG3’	379–389
*BCL-2*	rs11269260	Intron	TCTATCACCGATCATT/-	0.37	F 5’GCTTCCAGTTCCATCCATGT3’ R 5’CTCAGCGTGGTAGTGTTGGA3’	189–205
*TP53*	rs17880560	3’-Flanking	-/GCCGTG	0.21	F 5’CTGTGTGTCTGAGGGGTGAA3’ R 5’ATCCTGCCACTTTCTGATGG3’	400–406

### Analysis of ancestry informative markers (AIMs)

From a genetic perspective, the Brazilian population is one of the world’s most ethnically diverse, with a high degree of admixture. This is the result of the colonization process by many ancestral groups, including Native Americans, Europeans and Africans, over distinct time periods [25, 26]. Native Americans were the first settlers, coming from eastern Asia between 11,000 and 25,000 years BP [27, 28]. Europeans, mainly Portuguese, began colonization in the sixteenth century [29]. African populations were brought to Brazil from the 16th to the nineteenth century [26].

Population substructure may interfere in genetic studies of complex/multifactorial diseases by conferring spurious results [30]. To avoid misinterpretation in genotypic distribution due to population substructure, individual genomic ancestry was determined using a previously established panel of 61 autosomal AIMs that were standardized, validated, and showed significant variation in allele frequency across continental population groups from different geographic origins [30–32]. The allele frequencies of the 61 markers were obtained by multiplex PCR followed by capillary electrophoresis and fragment analysis. Then, individual proportions of European, African, and Native American genomic ancestries were estimated using the STRUCTURE v.2.3.3 software, which infers population admixture by estimating allele frequencies of the AIMs [33].

### Genotyping

Multiplex PCR was employed to simultaneously genotype the eight apoptosis markers, followed by capillary electrophoresis with fragment analysis. Each PCR reaction was performed in a final volume of 10 μL containing 1.0 μL of DNA (at a concentration of 10 ng/μL), 5.0 μL of QIAGEN Multiplex PCR Master Mix (QIAGEN, Hilden, Germany), 1.0 μL of Q-solution, 1.0 μL of primer mix and 2.0 μL of water, as previously established [22].

Amplification was conducted on the ABI Veriti thermal cycler (Thermo Fisher Scientific) using the following program: 95 °C for 15 min, followed by 35 cycles of 94 °C for 45 s, 60 °C for 90 s, and 72 °C for 1 min, with a final extension at 70 °C for 30 min. For fragment analysis, 1.0 μL of PCR product was added to 8.5 μL of HI-DI deionized formamide (Thermo Fisher Scientific) and 0.5 μL of GeneScan 500 LIZ pattern size standard (Thermo Fisher Scientific).

DNA fragments were separated using ABI PRISM 3130 genetic analyzer (Thermo Fisher Scientific) and analyzed with GeneMapper ID *v.3.2* software (Thermo Fisher Scientific). The multiplex PCR reaction and fragment analysis of the AIMs were similar to the protocol described above for the apoptosis panel.

### Statistical analyses

Statistical analyses were conducted in R software *v. 4.0.5* (R Development Core Team, 2021) adopting *P* ≤ 0.05 as statistically significant. Firstly, we calculated the Hardy–Weinberg Equilibrium (HWE) of each variant in the investigated groups using the chi-squared test corrected by the Bonferroni method. Ancestry indices were compared between groups using Mann–Whitney’s U test. While for the comparison of age, sex, and malaria infection history was used Student’s t-test, Pearson’s chi-squared and Fisher’s exact tests, respectively.

The association between the apoptosis INDEL variants, susceptibility to malaria, and parasite density were evaluated using a multivariate logistic regression to estimate Odds Ratio (OR) at 95% confidence intervals (CI). In the association analyses, genomic ancestry, age, sex, and infection history to control were considered as potential confounding effects.

## Results

### Clinical and demographic characteristics of the patients

Clinical and demographic metadata of malaria and control groups are summarized in Tables 2, 3. Malaria patients and controls presented significant differences in sex and genomic ancestry; all malaria groups were predominantly male, while female individuals were more frequent in the control group (*P. vivax vs* control group, *P* = 0.020; *P. falciparum vs* control group, *P* = 0.003; mixed infection *vs* control group, *P* = 0.013) (Table 2).

**Table 2:** Demographic characteristics of malaria and control groups

				*P-value*			
Variables	*Pf*^a^	*Pv*^b^	Mixed infection	CG^c^	*Pf vs* CG	*Pv* vs CG	Mixed infection *vs* CG
N	42	26	58	101	–	–	–
Age^d^	31.31 ± 13.22	31.10 ± 10.64	30.52 ± 10.02	31.60 ± 11.30	0.911	0.854	0.687
Sex (*M/F)^e^	75.0/25.0	68.4/31.60	67.6/32.40	48.5/51.50	**0.003**	**0.020**	**0.013**
Genomic Ancestry^f,g^							
European	0.529 ± 0.299	0.583 ± 0.272	0.587 ± 0.259	0.594 ± 0.201	0.354	0.890	0.766
African	0.242 ± 0.231	0.137 ± 0.169	0.161 ± 0.175	0.124 ± 0.124	**0.018**	0.392	**0.040**
Native American	0.235 ± 0.169	0.267 ± 0.169	0.263 ± 0.189	0.281 ± 0.180	0.302	0.976	0.602

The data are shown as mean ± standard deviation

*Pf*^a^, *Plasmodium falciparum*; *Pv*^b^, *Plasmodium vivax*; CG^c^, Control group; Student’s t-test^d^; Pearson’s chi-squared test^e^; Mann–Whitney test^f^; *M, male; F, female. The values highlighted in bold are statistically significant. The proportions of genomic ancestry were estimated using the STRUCTURE software^g^

**Table 3:** Demographic characteristics of malaria patients according to the infecting species

Variables	*Pf*^a^	*Pv*^b^	Mixed infection	*P-value*		
				*Pf* vs *Pv*	*Pf* vs Mixed infection	*Pv* vs Mixed infection
N	42	26	58			
Age^c^	31.31 ± 13.22	31.10 ± 10.64	30.52 ± 10.02	0.951	0.867	0.863
Sex (*M/F)^d^	75.0/25.0	68.4/31.60	67.6/32.40	0.253	0.320	1.000
Infection history^e^						
First infection (yes/no)	81.2/18.8	55.6/44.4	57.6/42.4	**0.008**	**0.035**	0.989
Genomic Ancestry^f,g^						
European	0.529 ± 0.299	0.583 ± 0.272	0.587 ± 0.259	0.561	0.373	0.980
African	0.242 ± 0.231	0.137 ± 0.169	0.161 ± 0.175	**0.030**	**0.048**	0.289
Native American	0.235 ± 0.169	0.267 ± 0.169	0.263 ± 0.189	0.283	0.301	0.745

*Pf*^a^, The data are shown as mean ± standard deviation

*Plasmodium falciparum*; *Pv*^b^, *Plasmodium vivax*; Student’s t-test^c^; Pearson’s chi-squared test^d^; Fisher’s exact test^e^; Mann–Whitney test^f^; *M, male; F, female. The values highlighted in bold are statistically significant. The proportions of genomic ancestry were estimated using the STRUCTURE software^g^

**Fig. 1:**
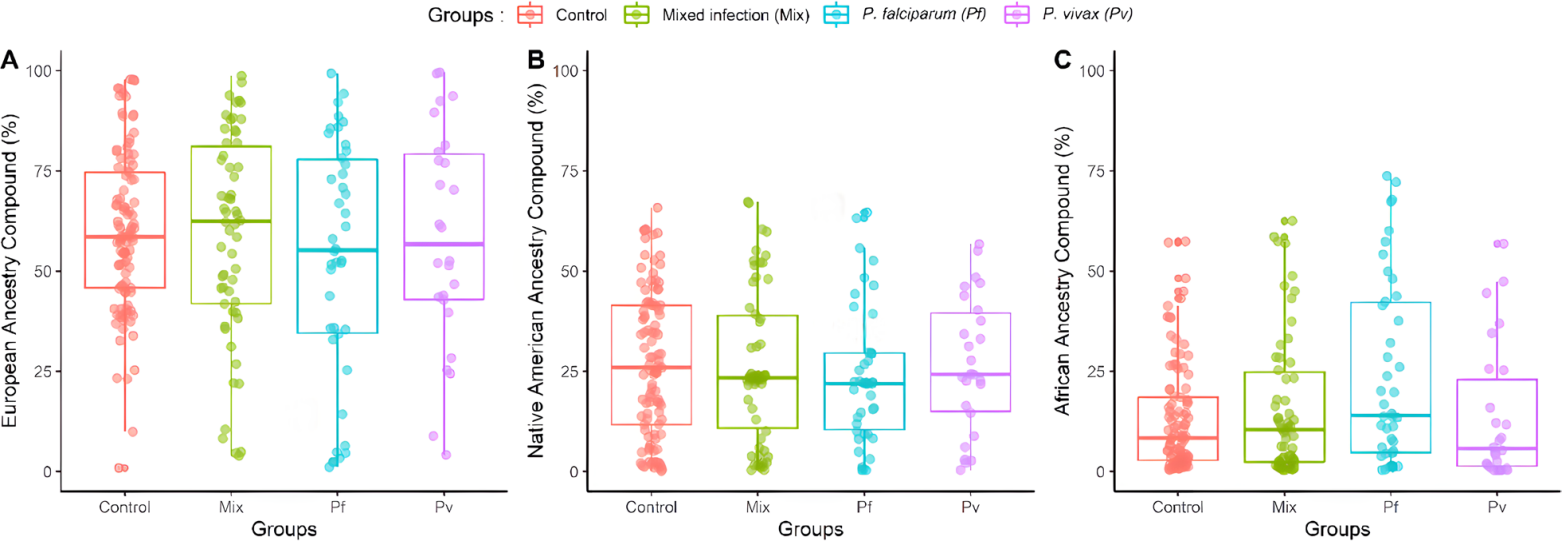
Distribution of ancestry proportions among the analysed groups. **A** European. **B** Native American. **C** African. Control, control group; MIX, mixed infection group; *Pf*, *Plasmodium falciparum* group*; Pv*, *Plasmodium vivax* group

Additionally, analysis of genetic ancestry showed that individuals infected with *P. falciparum* and with mixed infection had a higher proportion of African ancestry compared to the control group (*P. falciparum vs* control group, *P* = 0.018; mixed infection *vs* control group, *P* = 0.040) (Table 2). The distribution of genetic ancestries (European, African, and Native American) of the malaria patients and control groups is presented in Fig. 1.

### Analysis of the association between apoptosis variants and susceptibility to malaria

The allelic frequencies of variants analyzed in *P. falciparum*, *P. vivax*, mixed infection patients, and control group are represented in Additional file 3: Table S1. The distribution of all INDELs in the investigated populations followed HWE (Additional file 3: Table S1). Genotype association was performed using logistic regression adjusting for potential confounder factors and considering the following comparisons: (i) carriers of DEL/DEL genotype *vs* INS/DEL + INS/INS; and (ii) carriers of INS/INS genotype *vs* INS/DEL + DEL/DEL.

For *P. vivax*, significant associations with the risk for infection were not observed (Table 4). The analysis of *P. falciparum* and mixed infection individuals with control group showed that the INS/INS genotype of rs3834129 (*CASP8*) may increase the susceptibility to *P. falciparum* infection in approximately twofold (*P* = 0.038; OR = 1.867; 95% CI 0.736–3.725), while the DEL/DEL genotype of this marker indicated a significant genetic protection effect for *P. falciparum* infection (*P* = 0.049; OR = 0.446; 95% CI 0.185–0.944) (Table 5) and, in the mixed infection, the comparison also suggested genetic protection for *P. falciparum*, *P. vivax* and/or *P. malariae* infection (*P* = 0.026; OR = 0.545; 95% CI 0.281–0.996) (Table 6).

**Table 4:** Comparison of the genotypic distribution of the *P. vivax*-malaria and control group (CG)

Genotype	*Pv*^a^ (%)	CG^b^ (%)	*P*-value^c^	OR (95% CI)^d^	OR (95% CI)^e^
*FAS* (rs10562972)					
DEL/DEL	1 (3.8)	1 (0.9)	0.996	1.000 (0.000-∞)	1.051 (0.000-∞)
INS/DEL	1 (3.8)	24 (23.7)			
INS/INS	24 (92.4)	76 (75.4)	0.143	2.267 (0.455–5.109)	1.801 (0.860–4.259)
*FADD* (rs4197)					
DEL/DEL	10 (38.5)	45 (44.6)	0.457	0.855 (0.492–1.473)	0.802 (0.446–1.426)
INS/DEL	13 (50.0)	48 (47.5)			
INS/INS	3 (11.5)	8 (7.9)	0.365	1.169 (0.679–2.032)	1.420 (0.636–3.020)
*CASP8* (rs3834129)					
DEL/DEL	5 (19.2)	19 (18.8)	0.818	1.015 (0.534–1.675)	0.923 (0.458–1.780)
INS/DEL	14 (53.9)	43 (42.6)			
INS/INS	7 (26.9)	39 (38.6)	0.099	0.985 (0.536–1.870)	0.594 (0.312–1.095)
*CASP8* (rs59308963)					
DEL/DEL	7 (26.9)	29 (28.7)	0.805	0.947 (0.526–1.670)	0.925 (0.492–1.699)
INS/DEL	16 (61.6)	53 (52.5)			
INS/INS	3 (11.5)	19 (18.8)	0.142	1.055 (0.597–1.901)	0.551 (0.229–1.165)
*CASP9* (rs61079693)					
DEL/DEL	5 (19.2)	33 (32.7)	0.311	0.657 (0.349–1.189)	0.720 (0.375–1.338)
INS/DEL	18 (69.3)	44 (43.6)			
INS/INS	3 (11.5)	24 (23.7)	0.329	1.522 (0.841–2.860)	0.701 (0.330–1.390)
*CASP3* (rs4647655)					
DEL/DEL	17 (65.4)	51 (50.5)	0.176	1.466 (0.848–2.571)	1.499 (0.840–2.710)
INS/DEL	7 (26.9)	40 (39.6)			
INS/INS	2 (7.7)	10 (9.9)	0.702	0.682 (0.389–1.178)	0.853 (0.360–1.822)
*BCL2* (rs11269260)					
DEL/DEL	8 (30.8)	19 (18.8)	0.167	1.466 (0.813–2.597)	1.532 (0.828–2.800)
INS/DEL	11 (42.3)	45 (44.6)			
INS/INS	7 (26.9)	37 (36.6)	0.233	0.682 (0.385–1.230)	0.688 (0.368–1.254)
*TP53* (rs17880560)					
DEL/DEL	20 (77.0)	63 (62.4)	0.271	1.524 (0.856–2.786)	1.409 (0.773–2.634)
INS/DEL	5 (19.2)	34 (33.7)			
INS/INS	1 (3.8)	4 (3.9)	0.909	0.656 (0.359–1.167)	1.063 (0.324–2.768)

*Pv*^a^, *Plasmodium vivax*; CG^b^, Control group; *P*-value^c^ obtained through logistic regression adjusted by sex; Crude Odds Ratio (OR)^d^; Adjusted OR^e^

**Table 5:** Comparison of the genotypic distribution of the *P. falciparum*-malaria and control group (CG)

Genotype	*Pf*^a^ (%)	CG^b^ (%)	*P*-value^c^	OR (95% CI)^d^	OR (95% CI)^e^
*FAS* (rs10562972)					
DEL/DEL	3 (7.1)	1 (0.9)	0.993	1.000 (0.000-∞)	1.612 (0.000-∞)
INS/DEL	12 (28.6)	24 (23.7)			
INS/INS	27 (64.3)	76 (75.4)	0.392	0.535 (0.350–1.125)	0.780 (0.442–1.396)
*FADD* (rs4197)					
DEL/DEL	14 (33.3)	45 (44.6)	0.369	0.745 (0.467–1.181)	0.780 (0.453–1.333)
INS/DEL	23 (54.8)	48 (47.5)			
INS/INS	5 (11.9)	8 (7.9)	0.670	1.342 (0.847–2.139)	1.181 (0.534–2.491)
*CASP8* (rs3834129)					
DEL/DEL	5 (11.9)	19 (18.8)	**0.049**	**0.745 (0.413–1.293)**	**0.446 (0.185–0.944)**
INS/DEL	18 (42.9)	43 (42.6)			
INS/INS	19 (45.2)	39 (38.6)	**0.038**	**1.343 (0.773–2.420)**	**1.867 (0.736–3.725)**
*CASP8* (rs59308963)					
DEL/DEL	11 (26.2)	29 (28.7)	0.516	0.927 (0.568–1.497)	0.825 (0.457–1.459)
INS/DEL	20 (47.6)	53 (52.5)			
INS/INS	11 (26.2)	19 (18.8)	0.157	1.079 (0.668–1.760)	1.520 (0.847–2.712)
*CASP9* (rs61079693)					
DEL/DEL	11 (26.2)	33 (32.7)	0.762	0.889 (0.550–1.424)	0.917 (0.520–1.596)
INS/DEL	19 (45.2)	44 (43.6)			
INS/INS	12 (28.6)	24 (23.7)	0.077	1.124 (0.702–1.817)	1.652 (0.945–2.886)
*CASP3* (rs4647655)					
DEL/DEL	27 (64.3)	51 (50.5)	0.554	1.425 (0.902–2.262)	1.176 (0.686–2.022)
INS/DEL	14 (33.3)	40 (39.6)			
INS/INS	1 (2.4)	10 (9.9)	0.228	0.702 (0.442–1.108)	0.536 (0.161–1.326)
*BCL2* (rs11269260)					
DEL/DEL	7 (16.6)	19 (18.8)	0.385	0.921 (0.534–1.552)	0.734 (0.350–1.437)
INS/DEL	20(47.6)	45 (44.6)			
INS/INS	15 (35.8)	37 (36.6)	0.562	1.086 (0.644–1.872)	0.850 (0.488–1.463)
*TP53* (rs17880560)					
DEL/DEL	28 (66.6)	63 (62.4)	0.941	1.122 (0.707–1.792)	0.979 (0.569–1.692)
INS/DEL	13 (31.0)	34 (33.7)			
INS/INS	1 (2.4)	4 (3.9)	0.775	0.891 (0.558–1.414)	0.861 (0.271–2.186)

*Pf*^a^, *Plasmodium falciparum*; CG^b^, Control group; *P*-value^c^ obtained through logistic regression adjusted by sex and genomic ancestry (the values highlighted in bold are statistically significant); Crude Odds Ratio (OR)^d^; Adjusted OR^e^

**Table 6:** Comparison of genotypic distribution of the mixed infection-malaria and control group (CG)

Genotype	Mixed infection^a^ (%)	CG^b^ (%)	*P-*value^c^	OR (95% CI)^d^	OR (95% CI)^e^
*FAS* (rs10562972)					
DEL/DEL	1 (1.8)	1 (0.9)	1.000	1.000 (0.000-∞)	1.000 (0.000-∞)
INS/DEL	14 (21.1)	24 (23.7)			
INS/INS	43 (74.1)	76 (75.4)	0.746	0.991 (0.480–1.246)	1.087 (0.654–1.831)
*FADD* (rs4197)					
DEL/DEL	31 (53.5)	45 (44.6)	0.456	1.250 (0.834–1.876)	1.189 (0.753–1.878)
INS/DEL	22 (37.9)	48 (47.5)			
INS/INS	5 (8.6)	8 (7.9)	0.811	0.800 (0.533–1.199)	1.084 (0.547–2.083)
*CASP8* (rs3834129)					
DEL/DEL	6 (10.4)	19 (18.8)	**0.026**	**0.686 (0.401–1.139)**	**0.545 (0.281–0.996)**
INS/DEL	34 (58.6)	43 (42.6)			
INS/INS	18 (31.0)	39 (38.6)	0.201	1.458 (0.878–2.492)	1.732 (0.451–2.178)
*CASP8* (rs59308963)					
DEL/DEL	20 (34.5)	29 (28.7)	0.927	1.176 (0.772–1.792)	1.022 (0.631–1.644)
INS/DEL	26 (44.8)	53 (52.5)			
INS/INS	12 (20.7)	19 (18.8)	0.866	0.850 (0.558–1.296)	1.046 (0.608–1.775)
*CASP9* (rs61079693)					
DEL/DEL	13 (22.4)	33 (32.7)	0.176	0.734 (0.471–1.134)	0.709 (0.428–1.157)
INS/DEL	33 (56.9)	44 (43.6)			
INS/INS	12 (20.7)	24 (23.7)	0.794	1.362 (0.882–2.124)	0.933 (0.554–1.554)
*CASP3* (rs4647655)					
DEL/DEL	29 (50.0)	51 (50.5)	0.958	0.988 (0.659–1.482)	0.987 (0.625–1.560)
INS/DEL	25 (43.1)	40 (39.6)			
INS/INS	4 (6.9)	10 (9.9)	0.698	1.012 (0.675–1.518)	0.874 (0.430- 1.679)
*BCL2* (rs11269260)					
DEL/DEL	15 (25.9)	19 (18.8)	0.657	1.267 (0.808–1.984)	1.122 (0.665- 1.875)
INS/DEL	28 (48.2)	45 (44.6)			
INS/INS	15 (25.9)	37 (36.6)	0.092	0.789 (0.504–1.238)	0.655 (0.399- 1.062)
*TP53* (rs17880560)					
DEL/DEL	30 (51.7)	63 (62.4)	0.327	0.762 (0.506–1.144)	0.795 (0.502–1.259)
INS/DEL	23 (39.7)	34 (33.7)			
INS/INS	5 (8.6)	4 (3.9)	0.177	1.313 (0.874–1.974)	0.805 (0.805–3.184)

Mixed infection^a^, *Plasmodium* mixed infection malaria; CG^b^, Control group; *P*-value^c^ obtained through logistic regression adjusted by sex and genomic ancestry (the values highlighted in bold are statistically significant); Crude Odds Ratio (OR)^d^; Adjusted OR^e^

Within malaria subgroups, the INS/INS genotype of rs10562972 (*FAS*) was significantly more frequent among those infected with *P. vivax* than those with *P. falciparum* (*P* = 0.036; OR = 2.493; 95% CI 1.104–4.551) (Additional file 4: Table S2), suggesting that the carriers of this genotype have a 2.5-fold increased risk of developing malaria from *P. vivax* infection.

The frequency of the DEL/DEL genotype of rs17880560 (*TP53*) was also higher among those with mono-infection of *P. vivax* compared to those with mixed infection (*P* = 0.029; OR = 0.667; 95% CI 0.211–1.669) (Additional file 5: Table S3), indicating that carriers of this genotype may present some level of protection against infections by multiple species of *Plasmodium*. No marker showed an association in the comparison between mono-infected with *P. falciparum* and mixed infection (Additional file 6: Table S4).

### Analysis of the association of apoptosis panel with parasite density

The effect of variants on parasite density was also investigated. For this analysis, the measurements of parasite density were log-transformed (Log_10_) due to their skewed distribution. Moreover, the *P*-value and OR were adjusted for covariates that showed a significant effect on parasite density according to other studies, such as genetic ancestry, age, sex, and infection history [34–36]. Parasite density data containing geometric means, *P,* and OR values are presented in Additional file 7: Table S5 and Additional file 8: Table S6.

**Fig. 2:**
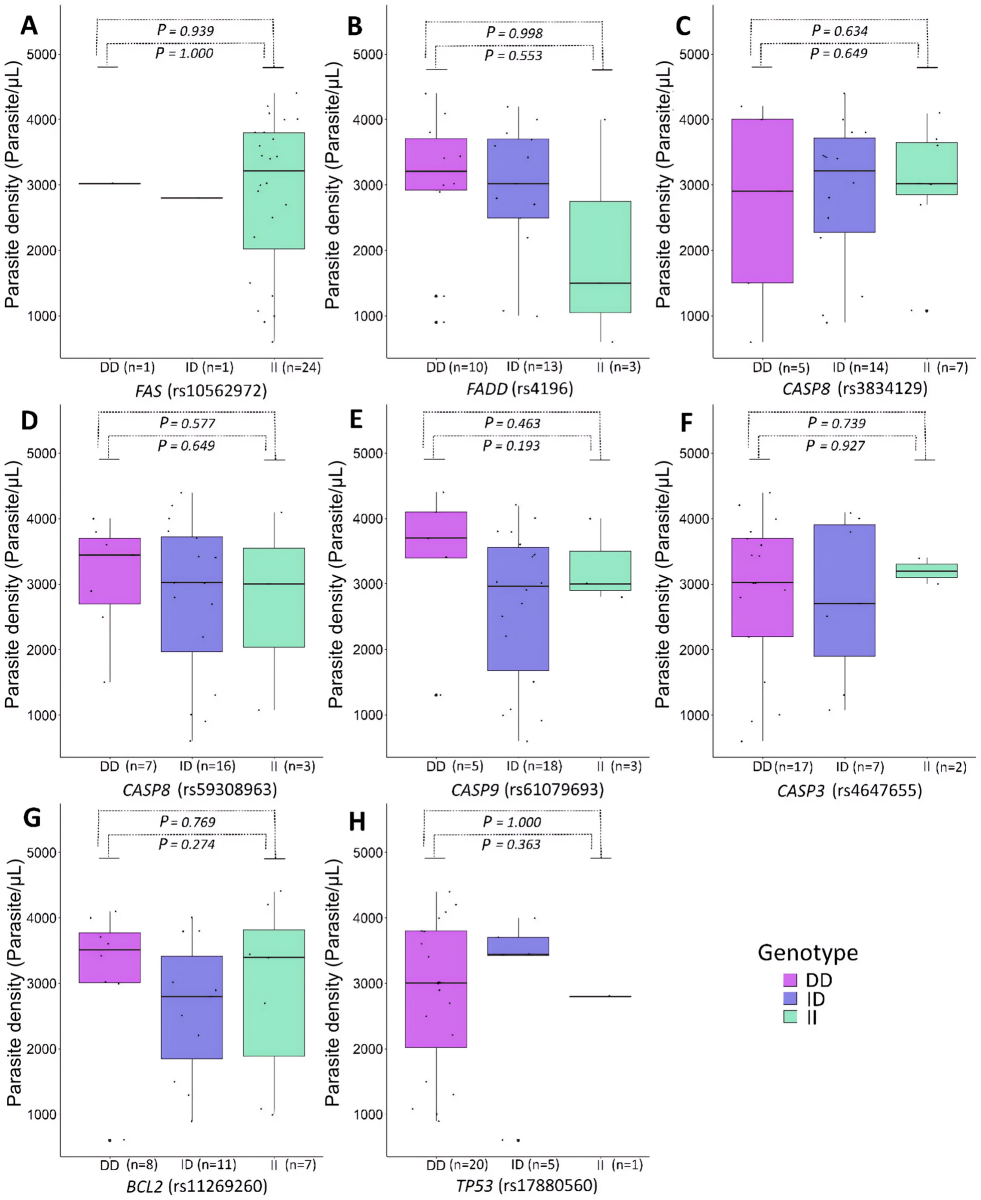
Association between apoptotic variants and *P. vivax* parasite density (parasites/ µL). **A** *FAS* (rs10562972). **B** *FADD* (rs4197). **C** *CASP8* (rs3834129). **D** *CASP8* (rs59308963). **(E)** *CASP9* (rs61079693). **F** *CASP3* (rs4647655). **G** *BCL2* (rs11269260). **H** *TP53* (rs17880560). DD, DEL/DEL genotype; ID, INS/DEL genotype; II, INS/INS genotype

**Fig. 3:**
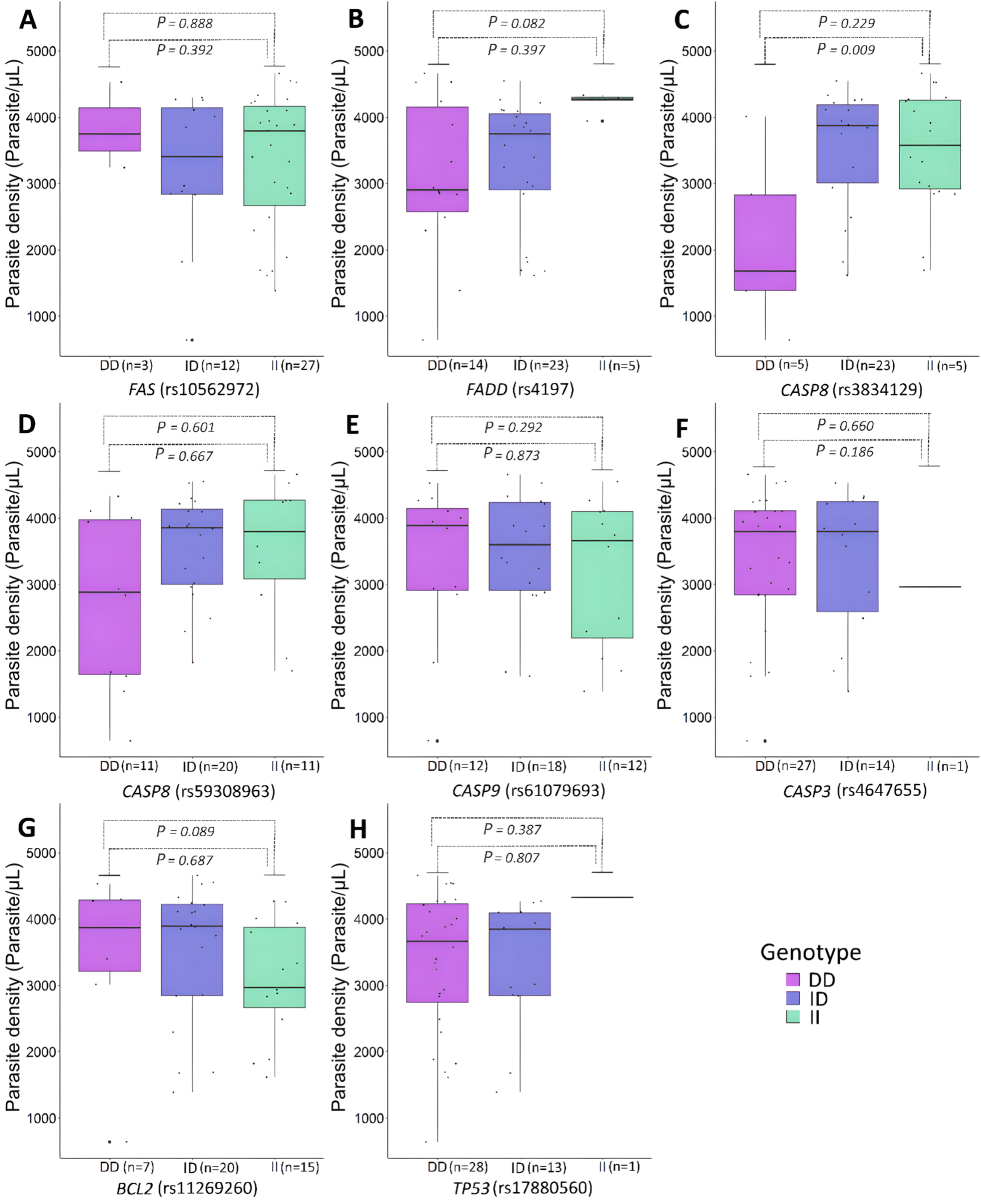
Association between apoptotic variants and *P. falciparum* parasite density (parasites/µL). **A** *FAS* (rs10562972). **B** *FADD* (rs4197). **C** *CASP8* (rs3834129). **D** *CASP8* (rs59308963). **E** *CASP9* (rs61079693). **F** *CASP3* (rs4647655). **G** *BCL2* (rs11269260). **H** *TP53* (rs17880560). DD, DEL/DEL genotype; ID, INS/DEL genotype; II, INS/INS genotype

In this analysis, no marker showed an association with the parasite density among individuals with *P. vivax*-malaria (Fig. 2A–H). Considering individuals with *P. falciparum*-malaria, the DEL/DEL genotype of the rs3834129 (*CASP8*) was associated with lower rates of parasite density (*P* = 0.009; OR = 0.383; 95% CI 0.113–1.295) (Fig. 3C).

## Discussion

Environmental conditions of the Amazon region are favourable to the circulation of parasites and their vectors, contributing to malaria being almost restricted to the Northern region of Brazil, where it is possible to find a significant percentage of mixed-species malaria infections [3, 5]. In addition to these environmental factors, host genetic profile may modulate the immune response and influence the susceptibility to *Plasmodium* infection, and in the outcome of malaria [37–40].

Throughout the years, important mechanisms for the immune response against infection and control of parasite density have been demonstrated by functional approaches and, in this context, apoptosis has been highlighted [12–14, 17]. The apoptosis of infected cells activates the innate and adaptive immune cells [12, 18]. Moreover, in acute malaria, apoptosis is critical in the depletion of immune cells [11, 15, 16].

Therefore, in this study, it was investigated the association between eight INDEL variants in apoptosis-related genes (*FAS*, *FADD*, *CASP8*, *BCL-2*, *CASP9*, *CASP3,* and *TP53*), and the infection by *P. falciparum*, *P. vivax,* and mixed infection, focusing on susceptibility to infection and parasite density. It was also investigated the potential influence of genomic ancestry in the susceptibility to malaria parasitosis considering the high admixture of the Brazilian Amazon population and that genomic ancestry has been associated with susceptibility to infectious diseases [35, 41–43].

Regarding these analyses, a significantly higher proportion of African ancestry among individuals with *P. falciparum* compared to the other groups was observed. Therefore, genomic ancestry seems to increase susceptibility to infection depending on the species of *Plasmodium* parasite. Similarly, a recent study also reported that increased African genomic ancestry was associated with higher risk for *P. falciparum* malaria in an admixed Colombian population [44].

Importantly, after correcting for African ancestry and other possible confounding factors in the association analysis between the investigated variants and susceptibility to malaria and parasite density, results showed significant associations between rs10562972 (*FAS*), rs3834129 (*CASP8*), and rs17880560 (*TP53*) and at least one of the malaria parameters, suggesting that these INDELs may influence the risk of malaria onset in the occurrence of *Plasmodium* infection as well as on parasite levels.

In addition, one of the most interesting findings presented here is related to genotypic frequencies of the rs3834129 variant. Located in the promoter region of the *CASP8* gene, the INS/INS genotype significantly increases the susceptibility to malaria caused by *P. falciparum*, while the DEL/DEL genotype reduces the chances for mono-infection by *P. falciparum* and for infection by multiple species. Moreover, the DEL/DEL genotype was associated with lower levels of parasite density.

These observations differ from the results obtained by Pinto and colleagues [42], in which the DEL/DEL genotype of this variant was a risk factor for infection by *Mycobacterium leprae*. However, they are similar to those observed in research with human papillomavirus (HPV), in which the DEL/DEL genotype was more frequent in non-infected individuals, and the INS allele was had higher frequencies in the HPV-positive group, while also associated with the risk of developing more severe clinical forms of HPV, especially in women of African ancestry [45, 46].

To date, the effects of the rs3834129 variant on apoptosis, specifically under an infectious condition, are still unknown. However, it has been demonstrated that this variant influences the surveillance of immune cells by suppressing the site where the Specific Protein 1 (SP1) transcription factor binds to the promoter, causing decreased expression of *CASP8* in T cells [47]. Indeed, it was later observed that, in experimental malaria, *CASP8* expression was associated with apoptosis of infected cells, as well as with the death of T cells and other immune cells in the spleen [17].

It is widely known that T cells play a crucial role in controlling parasite density by killing infected cells, and it occurs through the production of pro-inflammatory cytokines such as TNF and IFN-γ, in addition to the release of granzymes, perforins, and free radicals [48, 49]. Apoptosis of specific T cells for the malaria parasite impairs immune response and, consequently, the effector mechanism to control parasitic density [50]. Therefore, the investigated deletion of six nucleotides in the *CASP8* promoter may provide genetic protection to their carriers against infection and high parasite density by promoting the survival of T cells, increasing the efficiency of the immune response.

The other INDELs that showed a positive association in the present study are lesser known, and there is a lack of studies associating them with different types of diseases or other phenotypes. However, these variants seem to have a potential effect on protein expression, which can be reinforced by our work and should be further explored. Comparing infection by different *Plasmodium* species showed differences in the genotypic distribution of rs10562972 (*FAS*) and rs17880560 (*TP53*) variants. The INS/INS genotype of rs10562972 (*FAS*) was identified as a risk factor for malaria caused by *P. vivax* when compared to *P. falciparum*.

The *FAS* gene plays an important role in both the apoptosis of pathogen-infected cells [51] and in T cells under malaria infection [11]. However, there are still few reports of this apoptosis-related gene in malaria. Regardless, from a genetic point of view, the association between the single nucleotide polymorphism (SNP) rs22344767 and the susceptibility to infection by *P. vivax* in individuals from the Brazilian Amazon has been found [52], as well as a study in the Western African country of Ghana, that found an association of the SNP rs9658676 with protection for *P. falciparum*-associated severe malaria in Ghanaian children [53].

Another interesting finding was the observation of the DEL/DEL genotype of rs17880560 (*TP53*) as protective against mixed infection in the analysis *P. vivax* vs mixed infection. Currently, there are no studies reporting the effect of this variant on malaria. However, a functional study showed that *TP53*^−/−^ mice presented considerably higher parasite density compared to mice with overexpressed *TP53* [14]. This could mean that the presence of the altered INS allele possibly reduces the expression of *TP53*, decreasing the apoptosis of infected cells.

Interestingly, the results suggest that the intronic variants rs10562972 (*FAS*) and rs17880560 (*TP53*) are involved in the species-specific immune response to malaria, although it is not possible to establish its role in susceptibility yet, considering the biochemical effects of these mutations have not been assessed. Due to the great heterogeneity in the allelic distribution among populations [54], they may directly influence gene transcription.

Notably, this study has limitations such as a low sample size mainly in the *P. vivax*-infected group, which may influence statistical power. Despite these limitations, this study investigated a new panel of apoptosis markers with high potential to disrupt the activities of proteins involved in the extrinsic and intrinsic pathways of apoptosis. In addition, this study brings new information about the influence of these markers on different aspects of malaria, such as the risk of infection, including mixed infection and parasite density. It should be further noted that this cohort is composed of a Brazilian Amazon population. Similar studies should be carried out in other populations, considering that each admixed population presents a unique history of the formation process, and this should not be overlooked.

## Conclusion

In conclusion, identifying new molecular mechanisms involved in an ancient disease, such as malaria, can aid in the development of new drugs that will mitigate the impact of malaria on public health, considering the high rates of morbidity and mortality (about 600,000 deaths per year), especially in regions in South America and Africa. In this context, rs3834129 (*CASP8*) is a potential biomarker for susceptibility to *P. falciparum* and mixed infections, and it might influence parasite density. This study also highlights differences in the genotypic distribution of rs10562972 (*FAS*) in *P. vivax* vs *P. falciparum* and of rs17880560 (*TP53*) in the comparison between *P. vivax* and mixed infection, evidencing that genetic profile plays a role in species-specific susceptibility. Importantly, this is most likely the first study with this kind of approach for malaria infection in the Brazilian Amazon.

### Supplementary Information

**Additional file 1: Fig. S1.** Graphs of parasite density quantification estimated by RT-qPCR using the serial dilution of DNA from *P. vivax-infected* patients. (A) Threshold cycle detection for 10-fold dilutions of *P. vivax*. (B) Standard curve by DNA serial dilution containing 10^4^ to 10^-1^ *P. vivax *parasites per µL amplified in triplicate. The parameters of the standard curve are given by efficiency, coefficient of determination (r^2^), slope, and intercept values.

**Additional file 2: Fig. S2.** Graphs of parasite density quantification estimated by RT-qPCR using the serial dilution of DNA from *P. falciparum-*infected patients. (A) Threshold cycle detection for 10-fold dilutions of *P. vivax*. (B) Standard curve by DNA serial dilution containing 10^4^ to 10^-1^ *P. falciparum *parasites per µL amplified in triplicate. The parameters of standard curve are given by efficiency, coefficient of determination (r^2^), slope and intercept values.

**Additional file 3: Table S1.** Allele frequencies of INDELs for the eight investigated polymorphisms.

**Additional file 4: Table S2.** Comparison of genotypic distribution of *P. falciparum*- and *P. vivax*-malaria.

**Additional file 5: Table S3.** Comparison of genotypic distribution of and *P. vivax*-malaria and mixed infection.

**Additional file 6: Table S4.** Comparison of genotypic distribution of and *P. falciparum*-malaria and mixed infection.

**Additional file 7: Table S5.** Parasite density levels in *P. vivax* according to the genotypes.

**Additional file 8: Table S6.** Parasite density levels in *P. falciparum* according to the genotypes.

## Data Availability

The raw data supporting the conclusions of this research are available in the Figshare repository (https://doi.org/10.6084/m9.figshare.19203986).
