# Supplementary figures and images for: Association of apoptosis-related variants to malaria infection and parasite density in individuals from the Brazilian Amazon
Source: Malar J. 2023 Oct 4;22:295. doi: 10.1186/s12936-023-04729-6 (PMC10552311; doi:10.1186/s12936-023-04729-6)

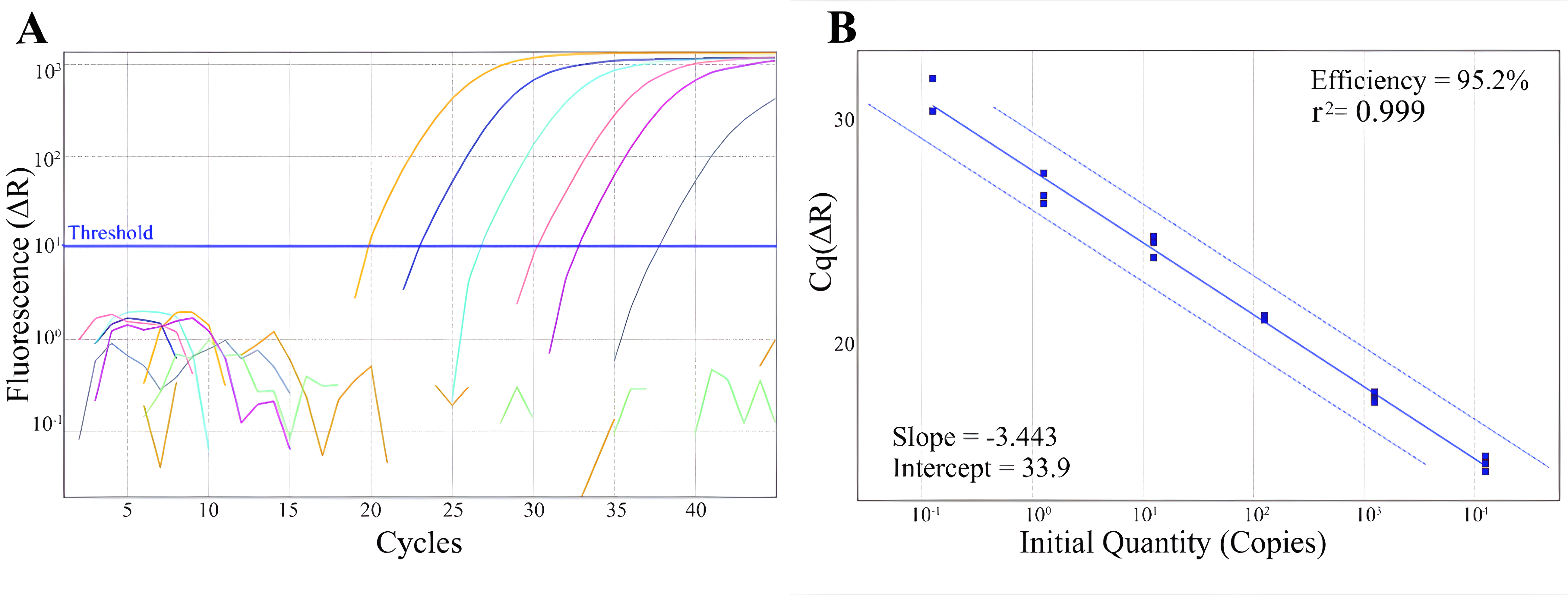

Supplement: Supplementary file 1 — Additional file 1: Fig. S1. Graphs of parasite density quantification estimated by RT-qPCR using the serial dilution of DNA from P. vivax-infected patients. (A) Threshold cycle detection for 10-fold dilutions of P. vivax. (B) Standard curve by DNA serial dilution containing 104 to 10-1 P. vivax parasites per µL amplified in triplicate. The parameters of the standard curve are given by efficiency, coefficient of determination (r2), slope, and intercept values. [file 12936_2023_4729_MOESM1_ESM.tif]

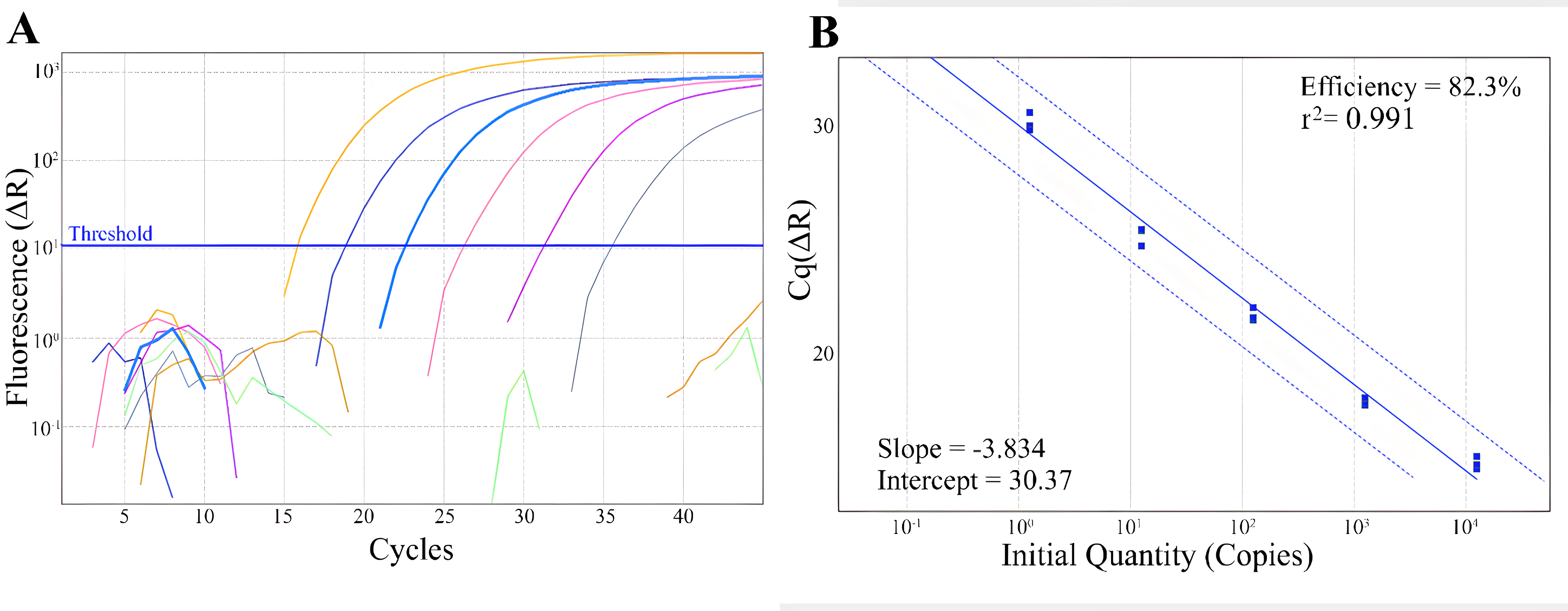

Supplement: Supplementary file 2 — Additional file 2: Fig. S2. Graphs of parasite density quantification estimated by RT-qPCR using the serial dilution of DNA from P. falciparum-infected patients. (A) Threshold cycle detection for 10-fold dilutions of P. vivax. (B) Standard curve by DNA serial dilution containing 104 to 10-1 P. falciparum parasites per µL amplified in triplicate. The parameters of standard curve are given by efficiency, coefficient of determination (r2), slope and intercept values. [file 12936_2023_4729_MOESM2_ESM.tif]
