# Supplementary material for: Association of apoptosis-related variants to malaria infection and parasite density in individuals from the Brazilian Amazon
Source: Malar J. 2023 Oct 4;22:295. doi: 10.1186/s12936-023-04729-6 (PMC10552311; doi:10.1186/s12936-023-04729-6)
Supplement: Supplementary file 3 — Additional file 3: Table S1. Allele frequencies of INDELs for the eight investigated polymorphisms. [file 12936_2023_4729_MOESM3_ESM.docx]

**Additional file 3**

| **Table S1.** Allele frequencies of INDELs for the eight investigated polymorphisms. | | | | |
| --- | --- | --- | --- | --- |
| **Gene (Polymorphism)** | ***Pv*^a^**  **(n=26)** | ***Pf*^b^**  **(n=42)** | **Mixed Infection^c^**  **(n=58)** | **Control Group^d^**  **(n=101)** |
| ***FAS* (rs10562972)** |  |  |  |  |
| INS | 0.943 | 0.786 | 0.862 | 0.881 |
| DEL | 0.057 | 0.214 | 0.138 | 0.119 |
| HWE^e^ | 0.495 | 0.712 | 0.499 | 0.773 |
| ***FADD* (rs4197)** |  |  |  |  |
| INS | 0.365 | 0.607 | 0.276 | 0.317 |
| DEL | 0.634 | 0.393 | 0.724 | 0.683 |
| HWE | 1.000 | 0.925 | 1.000 | 0.489 |
| ***CASP8* (rs3834129)** |  |  |  |  |
| INS | 0.538 | 0.667 | 0.604 | 0.599 |
| DEL | 0.462 | 0.333 | 0.396 | 0.401 |
| HWE | 1.000 | 1.000 | 1.000 | 0.395 |
| ***CASP8* (rs59308963)** |  |  |  |  |
| INS | 0.432 | 0.500 | 0.431 | 0.451 |
| DEL | 0.577 | 0.500 | 0.569 | 0.549 |
| HWE | 0.640 | 1.000 | 0.647 | 0.773 |
| ***CASP9* (rs61079693)** |  |  |  |  |
| INS | 0.461 | 0.500 | 0.491 | 0.455 |
| DEL | 0.538 | 0.500 | 0.509 | 0.545 |
| HWE | 0.495 | 0.944 | 0.495 | 0.231 |
| ***CASP3* (rs4647655)** |  |  |  |  |
| INS | 0.211 | 0.191 | 0.285 | 0.297 |
| DEL | 0.788 | 0.809 | 0.715 | 0.703 |
| HWE | 0.647 | 0.861 | 0.647 | 0.634 |
| ***BCL2* (rs11269260)** |  |  |  |  |
| INS | 0.481 | 0.595 | 0.500 | 0.589 |
| DEL | 0.519 | 0.405 | 0.500 | 0.411 |
| HWE | 0.673 | 0.934 | 0.677 | 0.416 |
| ***TP53* (rs17880560)** |  |  |  |  |
| INS | 0.135 | 0.178 | 0.285 | 0.208 |
| DEL | 0.865 | 0.822 | 0.715 | 0.792 |
| HWE | 0.667 | 0.688 | 0.667 | 1.000 |
| *Pv*^a^, *Plasmodium vivax*; *Pf*^b^, *Plasmodium falciparum*; Mixed infection^c^, *Plasmodium* mixed infection malaria; CG^d^, control group; HWE^e^, *P*-value for Hardy Weinberg equilibrium after Bonferroni correction. | | | | |
