# Supplementary material for: Association of apoptosis-related variants to malaria infection and parasite density in individuals from the Brazilian Amazon
Source: Malar J. 2023 Oct 4;22:295. doi: 10.1186/s12936-023-04729-6 (PMC10552311; doi:10.1186/s12936-023-04729-6)
Supplement: Supplementary file 4 — Additional file 4: Table S2. Comparison of genotypic distribution of P. falciparum- and P. vivax-malaria. [file 12936_2023_4729_MOESM4_ESM.docx]

**Additional file 4**

**Table S2.** Comparison of genotypic distribution of *P. falciparum*- and *P. vivax*-malaria.

| **Genotype** | ***Pf*^a^ (%)** | ***Pv*^b^ (%)** | ***P*-value^c^** | **OR (95%CI)^d^** | **OR (95%CI)^e^** |
| --- | --- | --- | --- | --- | --- |
| ***FAS* (rs10562972)** |  |  |  |  |  |
| DEL/DEL | 3 (7.1) | 1 (3.8) | 0.818 | 0.738 (0.225-1.996) | 0.878 (0.257-2.615) |
| INS/DEL | 12 (28.6) | 1 (3.8) |  |  |  |
| INS/INS | 27 (64.3) | 24 (92.4) | **0.036** | 1.355 (0.501-4.448) | 2.493 (1.104-4.551) |
| ***FADD* (rs4197)** |  |  |  |  |  |
| DEL/DEL | 14 (33.3) | 10 (38.5) | 0.680 | 1.147 (0.612-2.146) | 1.145 (0.602-2.178) |
| INS/DEL | 23 (54.8) | 13 (50.0) |  |  |  |
| INS/INS | 5 (11.9) | 3 (11.5) | 0.756 | 0.872 (0.466-1.635) | 0.876 (0.365-2.004) |
| ***CASP8* (rs3834129)** |  |  |  |  |  |
| DEL/DEL | 5 (11.9) | 5 (19.2) | 0.351 | 1.364 (0.647-2.874) | 1.443 (0.660-3.173) |
| INS/DEL | 18 (42.9) | 14 (53.9) |  |  |  |
| INS/INS | 19 (45.2) | 7 (26.9) | 0.074 | 0.733 (0.348-1.546) | 0.544 (0.278-1.046) |
| ***CASP8* (rs59308963)** |  |  |  |  |  |
| DEL/DEL | 11 (26.2) | 7 (26.9) | 0.761 | 1.022 (0.526-1.969) | 1.111 (0.559-2.199) |
| INS/DEL | 20 (47.6) | 16 (61.6) |  |  |  |
| INS/INS | 11 (26.2) | 3 (11.5) | 0.132 | 0.978 (0.508-1.901) | 0.552 (0.247-1.166) |
| ***CASP9* (rs61079693)** |  |  |  |  |  |
| DEL/DEL | 11 (26.2) | 5 (19.2) | 0.334 | 0.738 (0.367-1.449) | 0.706 (0.341-1.422) |
| INS/DEL | 19 (45.2) | 18 (69.3) |  |  |  |
| INS/INS | 12 (28.6) | 3 (11.5) | 0.125 | 1.355 (0.690-2.726) | 0.547 (0.247-1.147) |
| ***CASP3* (rs4647655)** |  |  |  |  |  |
| DEL/DEL | 27 (64.3) | 17 (65.4) | 0.751 | 1.030 (0.550-1.942) | 1.110 (0.580- 2.146) |
| INS/DEL | 14 (33.3) | 7 (26.9) |  |  |  |
| INS/INS | 1 (2.4) | 2 (7.7) | 0.368 | 0.971 (0.515-1.818) | 1.671 (0.566-5.636) |
| ***BCL2* (rs11269260)** |  |  |  |  |  |
| DEL/DEL | 7 (16.6) | 8 (30.8) | 0.318 | 1.592 (0.810-3.145) | 1.427 (0.708-2.876) |
| INS/DEL | 20 (47.6) | 11 (42.3) |  |  |  |
| INS/INS | 15 (35.8) | 7 (26.9) | 0.640 | 0.628 (0.318-1.235) | 0.853 (0.434-1.657) |
| ***TP53* (rs17880560)** |  |  |  |  |  |
| DEL/DEL | 28 (66.6) | 20 (77.0) | 0.292 | 1.357 (0.706-2.660) | 1.443 (0.743-2.850) |
| INS/DEL | 13 (31.0) | 5 (19.2) |  |  |  |
| INS/INS | 1 (2.4) | 1 (3.8) | 0.721 | 0.737 (0.376-1.416) | 1.263 (0.335-4.498) |
| *Pf*^a^, *Plasmodium falciparum*; *Pv*^b^, *Plasmodium vivax*; *P*-value^c^ obtained through logistic regression adjusted by infection history and genetic ancestry; Crude Odds Ratio (OR)^d^; Adjusted OR^e^. | | | | | |
