# Supplementary material for: Association of apoptosis-related variants to malaria infection and parasite density in individuals from the Brazilian Amazon
Source: Malar J. 2023 Oct 4;22:295. doi: 10.1186/s12936-023-04729-6 (PMC10552311; doi:10.1186/s12936-023-04729-6)
Supplement: Supplementary file 5 — Additional file 5: Table S3. Comparison of genotypic distribution of and P. vivax-malaria and mixed infection. [file 12936_2023_4729_MOESM5_ESM.docx]

**Additional file 5**

**Table S3.** Comparison of genotypic distribution of and *P. vivax*-malaria and mixed infection.

| **Genotype** | ***Pv*^a^ (%)** | **Mixed infection^b^ (%)** | ***P*-value^c^** | **OR (95%CI)^d^** |
| --- | --- | --- | --- | --- |
| ***FAS* (rs10562972)** |  |  |  |  |
| DEL/DEL | 1 (3.8) | 1 (1.8) | 0.996 | 87.788 (0.000-∞) |
| INS/DEL | 1 (3.8) | 14 (21.1) |  |  |
| INS/INS | 24 (92.4) | 43 (74.1) | 0.073 | 2.063 (0.976-4.879) |
| ***FADD* (rs4197)** |  |  |  |  |
| DEL/DEL | 10 (38.5) | 31 (53.5) | 0.204 | 0.683 (0.378-1.225) |
| INS/DEL | 13 (50.0) | 22 (37.9) |  |  |
| INS/INS | 3 (11.5) | 5 (8.6) | 0.677 | 1.180 (0.526-2.545) |
| ***CASP8* (rs3834129)** |  |  |  |  |
| DEL/DEL | 5 (19.2) | 6 (10.4) | 0.275 | 1.481 (0.725-2.990) |
| INS/DEL | 14 (53.9) | 34 (58.6) |  |  |
| INS/INS | 7 (26.9) | 18 (31.0) | 0.702 | 0.886 (0.474-1.631) |
| ***CASP8* (rs59308963)** |  |  |  |  |
| DEL/DEL | 7 (26.9) | 20 (34.5) | 0.490 | 0.805 (0.431-1.478) |
| INS/DEL | 16 (61.6) | 26 (44.8) |  |  |
| INS/INS | 3 (11.5) | 12 (20.7) | 0.306 | 0.683 (0.317-1.380) |
| ***CASP9* (rs61079693)** |  |  |  |  |
| DEL/DEL | 5 (19.2) | 13 (22.4) | 0.741 | 0.894 (0.455-1.711) |
| INS/DEL | 18 (69.3) | 33 (56.9) |  |  |
| INS/INS | 3 (11.5) | 12 (20.7) | 0.306 | 0.683 (0.317-1.380) |
| ***CASP3* (rs4647655)** |  |  |  |  |
| DEL/DEL | 17 (65.4) | 29 (50.0) | 0.190 | 1.485 (0.826-2.699) |
| INS/DEL | 7 (26.9) | 25 (43.1) |  |  |
| INS/INS | 2 (7.7) | 4 (6.9) | 0.896 | 1.059 (0.424-2.452) |
| ***BCL2* (rs11269260)** |  |  |  |  |
| DEL/DEL | 8 (30.8) | 15 (25.9) | 0.642 | 1.156 (0.622-2.126) |
| INS/DEL | 11 (42.3) | 28 (48.2) |  |  |
| INS/INS | 7 (26.9) | 15 (25.9) | 0.918 | 1.032 (0.549-1.914) |
| ***TP53* (rs17880560)** |  |  |  |  |
| DEL/DEL | 20 (77.0) | 30 (51.7) | **0.029** | 0.667 (0.211-1.669) |
| INS/DEL | 5 (19.2) | 23 (39.7) |  |  |
| INS/INS | 1 (3.8) | 5 (8.6) | 0.427 | 1.999 (1.085-3.780) |
| *Pv*^a^, *Plasmodium vivax*; Mixed infection^b^, *Plasmodium* mixed infection malaria; *P*-value^c^ obtained for logistic regression; Odds Ratio (OR)^d^. | | | | |
