# Supplementary material for: Association of apoptosis-related variants to malaria infection and parasite density in individuals from the Brazilian Amazon
Source: Malar J. 2023 Oct 4;22:295. doi: 10.1186/s12936-023-04729-6 (PMC10552311; doi:10.1186/s12936-023-04729-6)
Supplement: Supplementary file 6 — Additional file 6: Table S4. Comparison of genotypic distribution of and P. falciparum-malaria and mixed infection. [file 12936_2023_4729_MOESM6_ESM.docx]

**Additional file 6**

**Table S4.** Comparison of genotypic distribution of and *P. falciparum*-malaria and mixed infection.

| **Genotype** | ***Pf*^a^ (%)** | **Mixed infection^b^ (%)** | ***P*-value^c^** | **OR (95%CI)^d^** | **OR (95%CI)^e^** |
| --- | --- | --- | --- | --- | --- |
| ***FAS* (rs10562972)** |  |  |  |  |  |
| DEL/DEL | 3 (7.1) | 1 (1.8) | 0.993 | 1.000 (0.000-∞) | 1.013 (0.000-∞) |
| INS/DEL | 12 (28.6) | 14 (21.1) |  |  |  |
| INS/INS | 27 (64.3) | 43 (74.1) | 0.210 | 0.927 (0.653-1.348) | 0.714 (0.422-1.207) |
| ***FADD* (rs4197)** |  |  |  |  |  |
| DEL/DEL | 14 (33.3) | 31 (53.5) | 0.115 | 0.596 (0.357-0.988) | 0.589 (0.302-1.131) |
| INS/DEL | 23 (54.8) | 22 (37.9) |  |  |  |
| INS/INS | 5 (11.9) | 5 (8.6) | 0.651 | 1.678 (1.012-2.801) | 1.247 (0.487-3.291) |
| ***CASP8* (rs3834129)** |  |  |  |  |  |
| DEL/DEL | 5 (11.9) | 6 (10.4) | 0.806 | 1.085 (0.558-2.093) | 1.085 (0.557-2.093) |
| INS/DEL | 18 (42.9) | 34 (58.6) |  |  |  |
| INS/INS | 19 (45.2) | 18 (31.0) | 0.147 | 0.922 (0.478-1.792) | 1.455 (0.876-2.422) |
| ***CASP8* (rs59308963)** |  |  |  |  |  |
| DEL/DEL | 11 (26.2) | 20 (34.5) | 0.375 | 0.699 (0.412-1.320) | 0.788 (0.463-1.329) |
| INS/DEL | 20 (47.6) | 26 (44.8) |  |  |  |
| INS/INS | 11 (26.2) | 12 (20.7) | 0.520 | 1.269 (0.752-2.160) | 1.196 (0.691-2.070) |
| ***CASP9* (rs61079693)** |  |  |  |  |  |
| DEL/DEL | 11 (26.2) | 13 (22.4) | 0.484 | 1.210 (0.704-2.080) | 1.212 (0.706-2.077) |
| INS/DEL | 19 (45.2) | 33 (56.9) |  |  |  |
| INS/INS | 12 (28.6) | 12 (20.7) | 0.364 | 0.825 (0.481-1.416) | 1.285 (0.746-2.214) |
| ***CASP3* (rs4647655)** |  |  |  |  |  |
| DEL/DEL | 27 (64.3) | 29 (50.0) | 0.155 | 1.442 (0.872-2.396) | 1.442 (0.872-2.396) |
| INS/DEL | 14 (33.3) | 25 (43.1) |  |  |  |
| INS/INS | 1 (2.4) | 4 (6.9) | 0.309 | 0.693 (0.417-1.147) | 0.608 (0.201-1.477) |
| ***BCL2* (rs11269260)** |  |  |  |  |  |
| DEL/DEL | 7 (16.6) | 15 (25.9) | 0.271 | 0.726 (0.407-1.275) | 0.726 (0.406-1.275) |
| INS/DEL | 20 (47.6) | 28 (48.2) |  |  |  |
| INS/INS | 15 (35.8) | 15 (25.9) | 0.290 | 1.377 (0.784-2.457) | 1.325 (0.786-2.234) |
| ***TP53* (rs17880560)** |  |  |  |  |  |
| DEL/DEL | 28 (66.6) | 30 (51.7) | 0.135 | 1.477 (0.886-2.456) | 1.473 (0.888-2.456) |
| INS/DEL | 13 (31.0) | 23 (39.7) |  |  |  |
| INS/INS | 1 (2.4) | 5 (8.6) | 0.200 | 0.679 (0.407-1.126) | 0.539 (0.180-1.281) |
| *Pf*^a^, *Plasmodium falciparum*; Mixed infection^b^, *Plasmodium* mixed infection malaria; *P*-value^c^ obtained through logistic regression adjusted by infection history and genomic ancestry; Crude Odds Ratio (OR)^d^; Adjusted OR^e^. | | | | | |
