# Supplementary material for: Association of apoptosis-related variants to malaria infection and parasite density in individuals from the Brazilian Amazon
Source: Malar J. 2023 Oct 4;22:295. doi: 10.1186/s12936-023-04729-6 (PMC10552311; doi:10.1186/s12936-023-04729-6)
Supplement: Supplementary file 7 — Additional file 7: Table S5. Parasite density levels in P. vivax according to the genotypes. [file 12936_2023_4729_MOESM7_ESM.docx]

**Additional file 7**

**Table S5.** Parasite density levels in *P. vivax* according to the genotypes

| **Genotype** | ***Pv*^a^ (%)** | **Parasite density^b^ (range)** | ***P*-value^c^** | | **OR (95%CI)^d^** | **OR (95%CI)^e^** |
| --- | --- | --- | --- | --- | --- | --- |
| ***FAS* (rs10562972)** |  |  |  |  | |  |
| DEL/DEL | 1 (3.8) | 3020.6 | 1.000 | 0.138 (0.001-1.229) | | 0.704 (0.073-6.817) |
| INS/DEL | 1 (3.8) | 2800.0 |  |  | |  |
| INS/INS | 24 (92.4) | 2515.4 (600.0-4400.0) | 0.939 | 4.473 (0.714-72.744) | | 1.340 (0.237-7.572) |
| ***FADD* (rs4197)** |  |  |  |  | |  |
| DEL/DEL | 10 (38.5) | 2748.9 (900.0-4400.0) | 0.553 | 0.731 (0.157-3.154) | | 1.746 (0.609-5.007) |
| INS/DEL | 13 (50.0) | 2693.4 (1000.0-4200.0) |  |  | |  |
| INS/INS | 3 (11.5) | 1532.6 (600.0-4000.0) | 0.998 | 0.505 (0.120-1.936) | | 0.430 (0.095-1.948) |
| ***CASP8* (rs3834129)** |  |  |  |  | |  |
| DEL/DEL | 5 (19.2) | 2130.0 (600.0-4200.0) | 0.649 | 0.574 (0.131-2.302) | | 1.102 (0.260-4.673) |
| INS/DEL | 14 (53.9) | 2572.0 (900.0-4400.0) |  |  | |  |
| INS/INS | 7 (26.9) | 2824.1 (1074.6-4094.9) | 0.634 | 0.387 (0.076-1.609) | | 0.901 (0.235-3.458) |
| ***CASP8* (rs59308963)** |  |  |  |  | |  |
| DEL/DEL | 7 (26.9) | 2972.3 (1500.0-4000.0) | 0.649 | 0.322 (0.056-1.393) | | 0.859 (0.309-2.384) |
| INS/DEL | 16 (61.6) | 2409.2 (600.0-4400.0) |  |  | |  |
| INS/INS | 3 (11.5) | 2362.8 (1074.6-4094.9) | 0.577 | 0.358 (0.058-1.670) | | 1.114 (0.234-5.315) |
| ***CASP9* (rs61079693)** |  |  |  |  | |  |
| DEL/DEL | 5 (19.2) | 3117.7 (1300.0-4400.0) | 0.193 | 0.160 (0.020-0.821) | | 2.488 (0.873-7.093) |
| INS/DEL | 18 (69.3) | 2310.4 (600.0-4200.0) |  |  | |  |
| INS/INS | 3 (11.5) | 3226.8 (2800.0-4000.0) | 0.463 | 0.144 (0.007-0.940) | | 0.944 (0.241-3.707) |
| ***CASP3* (rs4647655)** |  |  |  |  | |  |
| DEL/DEL | 17 (65.4) | 2500.4 (600.0-4400.0) | 0.739 | 0.137 (0.003-1.024) | | 0.855 (0.314-2.327) |
| INS/DEL | 7 (26.9) | 2485.8 (1074.6-4094.9) |  |  | |  |
| INS/INS | 2 (7.7) | 3193.7 (3000.0-3400.0) | 0.927 | 1.529 (0.402-6.335) | | 1.123 (0.252-5.004) |
| ***BCL2* (rs11269260)** |  |  |  |  | |  |
| DEL/DEL | 8 (30.8) | 2825.3 (600.0-4094.9) | 0.274 | 0.514 (0.120-2.000) | | 1.666 (0.575-4.827) |
| INS/DEL | 11 (42.3) | 2376.7 (900.0-4000.0) |  |  | |  |
| INS/INS | 7 (26.9) | 2509.9 (1000.0-4400.0) | 0.769 | 0.308 (0.068-1.269) | | 0.861 (0.276-2.690) |
| ***TP53* (rs17880560)** |  |  |  |  | |  |
| DEL/DEL | 20 (76.9) | 2533.6 (900.0-4400.0) | 0.363 | 0.183 (0.004-1.464) | | 0.672 (0.210-2.143) |
| INS/DEL | 5 (19.2) | 2535.1 (600.0-4000.0) |  |  | |  |
| INS/INS | 1 (3.8) | 2800.0 | 1.000 | 2.640 (0.631-12.877) | | 0.801 (0.051-12.660) |
| *Pv*^a^, *Plasmodium vivax*; ^b^Parasite density tested with Log_10_ transformed values and presented as geometric means; *P-*value^c^ obtained after adjustment for ancestry, age, sex and infection history; Crude Odds Ratio (OR)^d^; Adjusted OR^e^. | | | | | | |
