# Supplementary material for: Association of apoptosis-related variants to malaria infection and parasite density in individuals from the Brazilian Amazon
Source: Malar J. 2023 Oct 4;22:295. doi: 10.1186/s12936-023-04729-6 (PMC10552311; doi:10.1186/s12936-023-04729-6)
Supplement: Supplementary file 8 — Additional file 8: Table S6. Parasite density levels in P. falciparum according to the genotypes. [file 12936_2023_4729_MOESM8_ESM.docx]

**Additional file 8**

**Table S6.** Parasite density levels in *P. falciparum* according to the genotypes

| **Genotype** | ***Pf*^a^ (%)** | **Parasite density^b^ (range)** | ***P*-value^c^** | **OR (95%CI)^d^** | **OR (95%CI)^e^** |
| --- | --- | --- | --- | --- | --- |
| ***FAS* (rs10562972)** |  |  |  |  |  |
| DEL/DEL | 3 (7.1) | 3804.2 (3242.0-4531.0) | 0.392 | 1.437 (0.380-5.588) | 1.596 (0.476-5.358) |
| INS/DEL | 12 (28.6) | 2929.4 (642.0-4300.0) |  |  |  |
| INS/INS | 27 (64.3) | 3168.5 (1388.0-4661.0) | 0.888 | 0.068 (0.001-0.675) | 1.039 (0.459-2.353) |
| ***FADD* (rs4197)** |  |  |  |  |  |
| DEL/DEL | 14 (33.3) | 2801.1 (642.0-4661.0) | 0.397 | 0.001 (2.738^e-7^-0.139) | 0.737 (0.348-1.562) |
| INS/DEL | 23 (54.8) | 3154.9 (1617.0-4551.0) |  |  |  |
| INS/INS | 5 (11.9) | 4218.4 (3944.0-4331.0) | 0.082 | 1.318 (0.350-10.013) | 2.217 (0.624-7.885) |
| ***CASP8* (rs3834129)** |  |  |  |  |  |
| DEL/DEL | 5 (11.9) | 1762.6 (642.0-4009.9) | **0.009** | 0.473 (0.112-1.797) | 0.383 (0.113-1.295) |
| INS/DEL | 18 (42.9) | 3390.2 (1617.0-4551.0) |  |  |  |
| INS/INS | 19 (45.2) | 3396.7 (1695.0-4661.0) | 0.228 | 2.324 (0.494-12.386) | 1.219 (0.561-2.647) |
| ***CASP8* (rs59308963)** |  |  |  |  |  |
| DEL/DEL | 11 (26.2) | 2415.6 (642.0-4331.0) | 0.667 | 0.303 (0.061-1.266) | 0.834 (0.373-1.864) |
| INS/DEL | 20 (47.6) | 3477.1 (1821.0-4551.0) |  |  |  |
| INS/INS | 11 (26.2) | 3386.7 (1695.0-4661.0) | 0.601 | 2.296 (0.581-9.793) | 1.205 (0.557-2.607) |
| ***CASP9* (rs61079693)** |  |  |  |  |  |
| DEL/DEL | 12 (28.6) | 3032.1 (642.0-4531.0) | 0.873 | 0.924 (0.235-3.493) | 1.016 (0.433-2.383) |
| INS/DEL | 18 (42.9) | 3347.7 (1617.0-4661.0) |  |  |  |
| INS/INS | 12 (28.6) | 2950.4 (1388.0-4551.0) | 0.292 | 0.571 (0.141-2.214) | 0.685 (0.336-1.396) |
| ***CASP3* (rs4647655)** |  |  |  |  |  |
| DEL/DEL | 27 (64.3) | 3136.5 (642.0-4661.0) | 0.186 | 1.358 (0.363-5.325) | 1.613 (0.783-3.325) |
| INS/DEL | 14 (33.3) | 3156.5 (1388.0-4531.0) |  |  |  |
| INS/INS | 1 (2.4) | 2966.0 | 0.660 | 0.235 (0.007-2.302) | 0.564 (0.065-4.927) |
| ***BCL2* (rs11269260)** |  |  |  |  |  |
| DEL/DEL | 7 (16.6) | 2987.5 (642.0-4531.0) | 0.687 | 1.590 (0.420-7.863) | 1.543 (0.363-6.557) |
| INS/DEL | 20(47.6) | 3339.2 (1388.0-4661.0) |  |  |  |
| INS/INS | 15 (35.8) | 2958.0 (1617.0-4270.0) | 0.089 | 0.323 (0.065-1.413) | 0.500 (0.217-1.149) |
| ***TP53* (rs17880560)** |  |  |  |  |  |
| DEL/DEL | 28 (66.6) | 3096.9 (642.0-4661.0) | 0.807 | 0.002 (1.732^e-14^-0.373) | 1.067 (0.460-2.474) |
| INS/DEL | 13 (31.0) | 3152.4 (1388.0-4270.0) |  |  |  |
| INS/INS | 1 (2.4) | 4331.0 | 0.387 | 1.714 (0.448-27.087) | 3.233 (0.441-23.683) |
| *Pf*^a^, *Plasmodium falciparum*; ^b^Parasite density tested with Log_10_ transformed values and presented as geometric means; *P-*value^c^ obtained after adjustment for ancestry, age, sex and infection history; Crude Odds Ratio (OR)^d^; Adjusted OR^e^. | | | | | |
